# New-onset or relapse of uveitis after rapid spreading of COVID-19 infection in China and risk factor analysis for relapse

**DOI:** 10.1186/s12886-024-03458-x

**Published:** 2024-04-26

**Authors:** Kaixuan Wang, Jiawen Li, Kailei Guo, Xiaomin Zhang

**Affiliations:** Tianjin Key Laboratory of Retinal Functions and Diseases, Tianjin Branch of National Clinical Research Center for Ocular Disease, Eye Institute and School of Optometry, Tianjin Medical University Eye Hospital, 251 Fu Kang Road, 300384 Tianjin, China

**Keywords:** Ocular inflammation, Uveitis, COVID-19, Methotrexate

## Abstract

**Background:**

The aim of this study was to report the clinical profile of new-onset and relapse of uveitis following rapid spreading of coronavirus disease 2019 (COVID-19) infection due to change of anti-COVID-19 policies in China and investigate potential risk factors for inflammation relapse.

**Methods:**

In this retrospective case-control study, patients with new-onset or a history of uveitis between December 23, 2022, and February 28, 2023, were included to assess the influence of COVID-19 infection on uveitis. Detailed information on demographic data, clinical characteristics, treatment measures, treatment response, and ocular inflammatory status before and after COVID-19 infection was collected.

**Results:**

This study included 349 patients with a history of uveitis. The uveitis relapse rate was higher (28.8%, *n* = 288) in those with COVID-19 infection than in patients without COVID-19 infection (14.8%, *n* = 61) (*P* = 0.024). Among the relapse cases, 50.8% experienced a relapse of anterior uveitis, while 49.2% had a relapse of uveitis involving the posterior segment. Multivariable regression analysis indicated a positive correlation between disease duration and uveitis relapse, while the last relapse exceeding one year before COVID-19 infection and the use of methotrexate during COVID-19 infection were negatively correlated with relapse of uveitis. Thirteen patients who developed new-onset uveitis following COVID-19 infection were included; among them, three (23.1%) had anterior uveitis and 10 (76.9%) had uveitis affecting the posterior segment. Regarding cases involving the posterior segment, four patients (30.8%) were diagnosed with Vogt-Koyanagi-Harada disease.

**Conclusions:**

COVID-19 infection increases the rate of uveitis relapse. Long disease duration is a risk factor, while time since the last relapse more than 1 year and methotrexate use are protective factors against uveitis relapse.

## Background

Viral infection can be a predisposing factor for autoreactivity and is involved in the mechanisms contributing to the initiation of autoimmune responses. Coronavirus disease 2019 (COVID-19) infection, caused by severe acute respiratory syndrome coronavirus 2 (SARS-CoV-2), may induce various immune-related diseases, such as Kawasaki-like disease, Guillain–Barré syndrome, Miller Fisher syndrome, systemic lupus erythematosus, rheumatoid arthritis, Sjögren’s syndrome, and uveitis [1, 2]. Since the onset of the global COVID-19 pandemic, the number of reported cases involving new-onset uveitis or its recurrence following COVID-19 infection has increased [3–6]. However, to date, attempts to identify potential links between these variables have been based predominantly on self-reported data on symptomatic uveitis from case reports or case series.

Despite earlier prevention strategies, COVID-19 rapidly spread in China following a change of anti-COVID-19 policies in December 2022. Several patients with a history of uveitis became infected within a short period of time, accompanied by an obvious increase in the recurrence of ocular inflammation among patients in our clinic. However, no studies to date have quantitively evaluated the link between these variables. Therefore, this study aimed to report the clinical profiles of patients experiencing new-onset or relapse of uveitis following the rapid spread of COVID-19 in a tertiary center in China, to compare the uveitis relapse rate between patients with and without COVID-19 infection, and to investigate potential risk factors for inflammatory relapse using multivariable regression analysis.

## Methods

### Selection of participants

This retrospective case-control study was conducted at Tianjin Medical University Eye Hospital, a tertiary ophthalmology center in China. This study adhered to the tenets of the Declaration of Helsinki and was approved by the institutional research ethics committee of Tianjin Medical University Eye Hospital (2023KY(L)-13), and informed consent signature exempted this study. Patients who visited the uveitis department between December 23, 2022, and February 28, 2023, were enrolled in the study. For those who had visited multiple times during this period, data from the first visit were used as reference points. The ocular inflammation was assessed by a uveitis specialist to ascertain whether it had relapsed since the prior visit or if it represented a new onset of uveitis. Additionally, the status and timing of their COVID-19 infection preceding the visit was investigated. Individuals with unknown inflammatory status prior to infection with COVID-19 were excluded. Individuals who had been infected with COVID-19 2 months ago or those whose COVID-19 infection status could not be ascertained were excluded.

As previously described [7], the criteria for determining COVID-19 infection status were as follows: [1] infected: individuals with a positive nucleic acid test, and those who met the clinical criteria and/or epidemiological criteria and had a positive result for the SARS-CoV-2 antigen rapid diagnostic test; [2] uninfected: individuals without COVID-19 symptoms and with a negative nucleic acid test or antigen rapid diagnostic test; and [3] infection status uncertain: individuals with a COVID-19 epidemiological history, with or without COVID-19 symptoms, but without results of antigen rapid diagnostic testing or nucleic acid testing.

### Data collection and assessment

The following data were collected for analysis: age, sex, disease duration, anatomical classification, specific diagnosis (etiology), associated systemic diseases, medications administered for uveitis treatment while infected with COVID-19, duration since the previous episode of recurrence, ocular condition at the time of new-onset uveitis or relapse episode, therapeutic interventions, and visual outcomes. The anatomical classification of uveitis was documented according to the Standardization of Uveitis Nomenclature (SUN) Working Group [8], and the SUN classification criteria [9] were used to diagnose uveitis etiology. Slit-lamp examinations were performed by a uveitis specialist. Best-corrected visual acuity (BCVA) was measured using a standard logarithmic visual acuity chart and converted to the logarithm of the minimum angle of resolution (logMAR) for statistical analysis. The following values were required for non-numeric visual acuity: counting fingers, 2.0; hand movements, 2.3; light perception, 2.7, and no light perception, 3.0 [10]. Additional examinations were performed as needed, including fundus photography, optical coherence tomography, and fundus fluorescein angiography. The necessary laboratory tests were performed to rule out other causes of intraocular inflammation in patients with new-onset uveitis. The ocular inflammation grading adhered to the SUN criteria [8]. In this study, the recurrence of inflammation at visit was determined according to a strict definition and was defined as the recurrence of iris nodules, keratic precipitates (KPs), an increase in the grading of the number anterior chamber cells and vitreous haze by ≥ 0.5 + when the grade was below 1 + and by ≥ 1 + when the grading was ≥ 1+, and the emergence of active fundus manifestations, such as macular edema, retinal or choroidal inflammation, serous retinal detachment, retinal hemorrhage, and retinal infiltration.

### Statistical analysis

Statistical analyses were performed using IBM SPSS Statistics (version 25.0; IBM Corp., Armonk, N.Y., USA). Continuous variables were presented as medians and interquartile ranges (IQR). Categorical data were expressed as frequencies and percentages. Normality was assessed based on histograms and quantile-quantile (Q-Q) plots, followed by one-way ANOVA and Wilcoxon signed-rank tests. Categorical data were compared using the chi-square or Fisher’s exact test. To determine the factors associated with uveitis relapse post-COVID-19 infection, binary logistic regression analysis was conducted. Parameters exhibiting *P* < 0.1 in the univariate analysis were carried forward into the multivariable regression analysis. Confidence intervals (CI) were computed at the 95% level, and *P* < 0.05 was deemed statistically significant.

## Results

### COVID-19 infection increased uveitis relapse

Data from 428 patients with a history of uveitis were reviewed. Of these 56 patients were excluded for having unknown inflammatory status before COVID-19 infection. Additionally, four and 19 patients were excluded for experiencing COVID-19 infection 2 months ago and for indeterminate COVID-19 statuses, respectively.

Ultimately, 349 patients with a history of uveitis were included, of whom 288 (82.5%) had been infected with COVID-19 and 61 (17.5%) had not. The participants’ median age was 31.0 years (IQR, 14.0–47.0 years), with 165 (47.3%) males and 184 (52.7%) females. The demographic characteristics and clinical features of uveitis in the infected and uninfected groups are presented in Table 1. Uveitis relapse occurred in 83 (28.8%) and nine (14.8%) patients with and without COVID-19, respectively (*P* = 0.024); thus, COVID-19 infection increased the uveitis relapse rate.

**Table 1:** Demographic and clinical characteristics of patients with a history of uveitis

Characteristics	COVID-19 infected patients	COVID-19 uninfected patients	P Value
	(*n* = 288, eyes = 482)	(*n* = 61, eyes = 102)	
Age (years) (M-IQR)	31.0 (14.0–46.0)	31.0 (15.0–55.0)	0.374
Gender (Male/female) (n, %)	130 (45.1)/158 (54.9)	35 (57.4)/26 (42.6)	0.062
Course of disease (years) (M-IQR)	2.50 (1.17–4.90)	3.4 (1.54-6.00)	0.033
BCVA^a^ (logMAR, M-IQR)	0.00 (0.00-0.22)	0.10 (0.00-0.30)	0.100
The anterior chamber cell grade recorded as 0^b^ (eyes, %)	398 (82.6)	82 (80.4)	0.601
Infective uveitis (n, %)	9 (3.1)	4 (6.6)	0.361
Anatomical classification (n, %)			
Anterior uveitis	30 (10.4)	11 (18.0)	0.093
Intermediate, posterior or panuveitis	258 (89.6)	50 (82.0)	
Uveitis-related systemic diseases (n, %)			
Vogt-Koyanagi-Harada disease	47 (16.3)	4 (6.6)	0.05
Behcet’s disease	26 (9.0)	5 (8.2)	0.836
Ankylosing Spondylitis	19 (6.6)	2 (3.3)	0.488
Other diseases^c^	16 (5.6)	5 (8.2)	0.623

M, median; IQR, interquartile range;

^a^ BCVA at the last visit;

^b^ The anterior chamber cell grade recorded as 0 at the last visit

^c^ Other Uveitis-related systemic diseases included multiple sclerosis, sjogren’s syndrome, rheumatoid arthritis, psoriasis, juvenile idiopathic arthritis, sarcoidosis, blau syndrome, kawasaki disease

### Profile and treatment of COVID-19-infected patients

Among the 288 patients with a history of uveitis who were infected with COVID-19, the onset of COVID-19 occurred from early December to early January, peaking in mid-December, 2022. Eighty-three patients (126 eyes) experienced relapse post-COVID-19 infection, whereas 205 (356 eyes) did not; the etiological classifications of uveitis in these patients are presented in Table 2. Idiopathic uveitis was the most prevalent etiology, affecting 165 (57.3%) patients, followed by Vogt-Koyanagi-Harada (VKH) disease in 47 (16.3%) patients and Behçet’s disease-related uveitis in 26 (9.0%) patients. Comprehensive clinical data and information on the therapeutic agents administered during COVID-19 infection in the groups who did and did not experience relapse are summarized in Table 3. Among the 83 patients (126 eyes) who experienced uveitis relapse post-COVID-19 infection, 40 (48.2%) and 43 (51.8%) exhibited monocular and bilateral relapse, respectively. Only relapse of anterior uveitis occurred in 64 eyes (50.8%) and was characterized by an increase in anterior chamber cells, iris nodule emergence, and keratic precipitates (KPs). Relapse involving posterior segment uveitis was present in 62 eyes (49.2%) and was marked by increased vitreous inflammatory haze and the emergence of active fundus manifestations. The median best-corrected visual acuity (BCVA) at relapse was 0.10 (IQR, 0.00–0.30) and the median intraocular pressure was 14.7 mmHg (IQR, 12.2–17.4 mmHg). Among the patients who experienced uveitis relapse, 64 (77.1%) were treated with topical corticosteroid eye drops, and two (2.4%) underwent peribulbar triamcinolone acetonide injections. Nine (10.8%) and 35 (42.2%) patients with uveitis relapse involved the posterior segment and required intravitreal steroid implants and enhanced systemic therapy, respectively. The systemic therapy involved the escalation of dosages, extension of the duration of drug administration, and alterations or augmentation of the types of anti-inflammatory medications administered. Eleven patients (13.3%) were lost to follow-up post-relapse; the remaining 72 patients (86.7%) were followed up for a median duration of 5 months (IQR, 3–6 months) post-relapse and responded favorably to treatment; regardless of the uveitis type, inflammation improved or resolved. The median BCVA at the final follow-up was 0.00 (IQR, 0.00–0.10), and the median intraocular pressure was 14.2 mmHg (IQR, 12.4–17.1 mmHg).

**Table 2:** Etiological classification of patients with a history of uveitis infected with COVID-19

Etiology (n, %)	Total (*n* = 288)	Relapsed patients	Non-relapsed
		(*n* = 83)	patients (*n* = 205)
Vogt-Koyanagi-Harada disease	47 (16.3)	14 (16.9)	33 (16.1)
Behcet’s disease-related uveitis	26 (9.0)	4 (4.8)	22 (10.7)
Ankylosing spondylitis associated uveitis	19 (6.6)	9 (10.8)	10 (4.9)
Other systemic diseases-related uveitis^a^	16 (5.6)	6 (7.2)	10 (4.9)
Viral anterior uveitis	4 (1.4)	1 (1.2)	3 (1.5)
Fuchs’ heterochromic iridocyclitis	4 (1.4)	1 (1.2)	3 (1.5)
Cytomegalovirus retinitis	2 (0.7)	0	2 (1.0)
Acute retinal necrosis syndrome	2 (0.7)	0	2 (1.0)
Posner-Schlossman Syndrome	2 (0.7)	0	2 (1.0)
Toxoplasmosis	1(0.3)	0	1 (0.5)
Idiopathic uveitis	165 (57.3)	48 (57.8)	117 (57.1)

^a^ Other systemic diseases included multiple sclerosis, sjogren’s syndrome, rheumatoid arthritis,

psoriasis, juvenile idiopathic arthritis, sarcoidosis, blau syndrome, kawasaki disease

**Table 3:** Comprehensive clinical data, medications employed and risk factors for relapse in both cohorts

Parameters	Relapsed patients (*n* = 83, eyes = 126)	Non-relapsed patients (*n* = 205, eyes = 356)	Univariate		Multivariate	
			OR (95% CI)	P Value	OR (95% CI)	P Value
Age (years) (M-IQR)	31 (15–48)	31 (14–44)	1.006 (0.992–1.020)	0.398	NA	NA
Gender (male/female) (n, %)	32 (38.6)/51(61.4)	98 (47.8)/107(52.2)	1.460 (0.868–2.455)	0.154	NA	NA
Course of disease (years) (M-IQR)	3.25 (1.58–5.33)	2.17 (1.08-4.00)	1.054 (0.995–1.116)	0.075	1.082 (1.016–1.151)	0.013
BCVA^a^ (logMAR, M-IQR)	0.00 (0.00-0.22)	0.00 (0.00-0.15)	0.831 (0.518–1.333)	0.443	NA	NA
The anterior chamber cell grade recorded as 0^b^ (eyes, %)	103 (81.7)	295 (82.9)	0.926 (0.545–1.572)	0.776	NA	NA
Infectious uveitis (n, %)	1 (1.2)	8 (2.2)	3.330 (0.410-27.051)	0.260	NA	NA
Anatomical classification (n, %)						
Anterior uveitis	10 (12.0)	20 (9.8)	1	0.565	NA	NA
Intermediate, posterior or panuveitis	73 (88.0)	185 (90.2)	0.789 (0.352–1.767)	NA	NA	NA
Uveitis-related systemic diseases (n, %)						
Vogt-Koyanagi-Harada disease	14 (16.9)	33 (16.1)	0.946 (0.477–1.875)	0.873	NA	NA
Behcet’s disease	4 (4.8)	22 (10.7)	2.374 (0.792–7.115)	0.123	NA	NA
Ankylosing Spondylitis	9 (10.8)	10 (4.9)	0.422 (0.165–1.079)	0.072	0.406 (0.145–1.133)	0.085
Other diseases^c^	6 (7.2)	10 (4.9)	0.658 (0.231–1.873)	0.433	NA	NA
Other systemic diseases (n, %)						
Hypertension	9 (10.8)	12 (5.9)	0.511 (0.207–1.264)	0.146	NA	NA
Diabetes	2 (2.4)	9 (4.4)	1.860 (0.393–8.796)	0.434	NA	NA
Malignancy	1 (1.2)	3 (1.5)	1.218 (0.125–11.879)	0.865	NA	NA
Time since the last relapse > 1 year (n, %)	20 (24.1)	82 (40.0)	0.476 (0.268–0.847)	0.012	0.335 (0.177–0.632)	0.001
Systemic medication^d^(n, %)						
Prednisone	36 (43.4)	91 (44.4)	0.960 (0.574–1.605)	0.875	NA	NA
Immunosuppressants	31 (37.3)	103 (50.2)	0.590 (0.350–0.995)	0.048	NA	NA
Cyclosporin A	4 (4.8)	3 (1.5)	3.409 (0.746–15.578)	0.114	NA	NA
Methotrexate	22 (26.5)	82 (40.0)	0.541 (0.308–0.949)	0.032	0.516 (0.288–0.926)	0.026
Mycophenolate mofetil	8 (9.6)	19 (9.3)	1.044 (0.438–2.489)	0.922	NA	NA
Azathioprine	0	1 (0.5)	0	1.000	NA	NA
Adalimumab	22 (26.5)	72 (35.1)	0.666 (0.378–1.173)	0.159	NA	NA
Types of systemic medications (n, %)		205				
0	32 (38.6)	58 (28.3)	1	0.349	NA	NA
1	20 (24.1)	55 (26.8)	0.659 (0.337–1.287)	NA	NA	NA
2	19 (22.9)	66 (32.2)	0.558 (0.288–1.081)	NA	NA	NA
3 types and above	12 (14.5)	26 (12.7)	0.738 (0.324–1.682)	NA	NA	NA
Self-delayed Medications^e^ (n, %)	19 (22.9)	39 (19.0)	1.264 (0.680–2.348)	0.459	NA	NA
Vaccination (n, %)	76 (91.6)	192 (93.7)	1.360 (0.523–3.540)	0.528	NA	NA

M, median; IQR, interquartile range; NA, not applicable

^a^ BCVA at the last visit before the COVID-19 infection

^b^ The anterior chamber cell grade recorded as 0 at the last visit before the COVID-19 infection

^c^ Other Uveitis-related systemic diseases included multiple sclerosis, sjogren’s syndrome, rheumatoid arthritis, psoriasis, juvenile idiopathic arthritis, sarcoidosis, blau syndrome, kawasaki disease

^d^ Systemic medication utilized for the treatment of uveitis during COVID-19 infection

^e^ Self-delayed medications for uveitis during COVID-19 infection

### Risk factors for uveitis relapse post-COVID-19 infection

The risk factors for uveitis relapse following COVID-19 infection are presented in Table 3. The univariate analysis revealed that the disease course (odds ratio [OR], 1.054; *P* = 0.075), ankylosing spondylitis (OR, 0.422; *P* = 0.072), length of time since the last relapse episode exceeding one year (OR, 0.476; *P* = 0.012), and methotrexate use during COVID-19 infection (OR, 0.541; *P* = 0.032) were associated with uveitis relapse. The multivariable regression analysis indicated that the disease course (OR, 1.082; *P* = 0.013), length of time since the last relapse episode exceeding one year (OR, 0.335; *P* = 0.001), and methotrexate use during COVID-19 infection (OR, 0.516; *P* = 0.026) remained independent related factors. The longer the disease duration, the higher the likelihood of uveitis relapse. Patients with over one year since the last relapse episode exhibited a lower risk of uveitis relapse than those who had experienced relapse within the previous year. Additionally, patients who received methotrexate during COVID-19 infection demonstrated a lower risk of uveitis relapse than those who did not.

### New-onset uveitis post-COVID-19 infection

In total, 13 patients, including two men (15.4%) and 11 women (84.6%) experienced new-onset uveitis post-COVID-19 infection, with a median age of 53 years (IQR, 18–59 years). The median interval between COVID-19 infection and uveitis onset was 20.0 days (IQR, 7.5–29.0 days). Table 4 summarizes the baseline characteristics, clinical manifestations, and treatment outcomes in these patients. Among them, three (23.1%) presented with anterior uveitis, 10 (76.9%) exhibited uveitis involving the posterior segment, of which four (30.8%) were diagnosed with VKH disease. All three patients with anterior uveitis achieved inflammation resolution and discontinued medication. Patients with uveitis affecting the posterior segment attained remission of inflammation and tapered medication, except for two patients who were lost to follow-up and one who declined treatment with oral medications.

**Table 4:** Demographic characteristics and specific clinical information of patients with new onset uveitis following COVID-19 infection

Patient No.	Sex	Age	Time interval of onset (days)	Etiology	eye	History of uveitis-related systemic disease	History of ocular disease	Initial visit			Treatment	Follow-up time (months)	Final visit		
								BCVA (Log-MAR)	IOP	Ocular examinations			BCVA (Log-MAR)	IOP	Ocular examinations
1	F	57	26	VKH	OU	-	Cataracts	0.8/0.7	16.2/19.3	OU: AC cells grade 2+; Dust-like KPs; VH grade 2+; Optic disc edema; ERD; Choroiditis	Steroid eye drops; Mydriatic eye drops; Oral prednisone	6	0.1/0.1	15.7/16.4	OU: AC cells grade 0; VH grade 0; Resolution of sub-retinal fluids
2	F	49	42	VKH	OU	-	-	0.3/1.0	13.6/13.6	OU: Optic disc edema ; ERD; Choroiditis	Oral prednisone	1.5	0.2/0.4	14.9/13.0	OU: Resolution of subretinal fluids
3	F	76	30	VKH	OU	-	Cataracts	0.8/1.0	23.2/20.9	OU: AC cells grade 3+; VH grade 1+; Optic disc edema; ERD; Choroiditis	Steroid eye drops; Mydriatic eye drops; Oral prednisone	1	0.5/0.5	12.7/14.9	OU: Resolution of subretinal fluids
4	M	23	10	VKH	OU	-	FS-LASIK Postoperative	0.0/0.0	17.7/16.3	OU: Optic disc edema; ERD; Choroidi-tis	Oral prednisone	6.5	0.0/0.0	14.8/13.4	OU: Resolution ofsubretinal fluids
5	F	7	28	JIA-related anterior uveitis	OS	JIA	-	0.0	9.7	OS: AC cells grade 2+; Mutton-fat KPs	Steroid eye drops; Mydriatic eye drops;	2	0.0	11.8	OS: AC cells grade 0
6	F	69	1	Idiopathic anterior uveitis	OD	-	AMD	1.3	16.4	OD: AC cells grade 0.5+	Steroid eye drops	2	1.3	18.6	OD: AC cells grade 0
7	F	58	20	Idiopathic anterior uveitis	OS	-	-	0.1	18.5	OS: AC cells grade 2+;	Steroid eye drops; Mydriatic eye drops;	2	0.0	19.8	OS: AC cells grade 0
8	M	12	8	pediatric intermedia-te uveitis	OU	-	-	0.0/0.0	16.2/12.8	OU: AC cells grade2+; VH grade 0.5+;Peripheral retinal capillary leakage	Steroid eye drops; Mydriatic eye drops; Oral methotrexate; ad-alimumab	5	0.0/0.0	16.8/15.9	OU: AC cells grade 0;VH grade 0
9	F	53	10	Idiopathic posterior uveitis	OS	-	-	0.0	17.5	OS: VH grade 0.5+; Chorioretinitis	Oral prednisone	No follow-up visits	-	-	-
10	F	56	20	Idiopathic panuveitis	OS	-	Cataracts	0.0	13.4	OS: AC cells grade 1+; VH grade 0.5+; Chorioretinitis	Patient refused oral corticostero-ids; Steroid eye drops; Mydriatic eye drops;	1.5	0.0	18.3	OS: AC cells grade 0;VH grade 0.5+; Chorioretinitis
11	F	60	3	Idiopathic panuveitis	OD	-	-	0.4	17.3	OD: iris posterior synechia; VH grade 2+; diffuse retinal vasculitis;	Steroid eye drops; Mydriatic eye drops; Oral prednisone	5	0.0	18.7	OD: VH grade 0; Reduced retinal vascular leakage
12	F	39	34	Idiopathic panuveitis	OS	-	-	0.4	9.6	OS: AC cells grade 3+; VH grade 2+; Chorioretinitis	Steroid eye drops; Mydriatic eye drops; Oral prednisone; Oral azathioprine	6	0.0	13.2	OS: AC cells grade 0;VH grade 0
13	F	13	7	pediatric panuveitis	OU	-	OS: Amblyopia	0.1/0.7	11.1/10.5	OD: AC cells grade2+; VH grade 3+. OS: AC cells grade1+; VH grade 2+. OU: Mutton-fat KPs; iris posterior synechia; CME	Steroid eye drops; Mydriatic eye drops; Oral prednisone; Oral methotrexate	No follow-up visits	-	-	-

JIA, Juvenile Idiopathic Arthritis; FS-LASIK, Femtosecond Laser-Assisted in Situ Keratomileusis; AMD, Age-related Macular Degeneration; BCVA, best-corrected visual acuity; AC, anterior chamber; KPs, keratic precipitates; VH, vitreous haze; ERD, exudative retinal detachment; CME, cystoid macular edema; OD, oculus dexter; OS, oculus sinister; OU, oculi uniter

## Discussion

Despite the observed heterogeneity in the types of new-onset or recurrent uveitis cases post-COVID-19 infection, responses to conventional treatments remained favorable. This study confirmed that COVID-19 infection increased the uveitis relapse rate, which increased with the disease duration. In contrast, the relapse risk was reduced in patients who received methotrexate while infected and in those whose last relapse episode occurred more than one year before COVID-19 infection.

A previous study has reported that 53.8% of uveitis cases occurring post-COVID-19 infection involved anterior uveitis, and 60.0% of newly diagnosed cases involved anterior uveitis [11]. However, in our study, anterior uveitis accounted for 23.1% of new-onset uveitis and VKH accounted for 30.8%. In these relapse cases, 50.8% and 49.2% involved anterior and posterior uveitis relapse, respectively, with no known type of uveitis showing a higher probability of relapse. Cases of VKH disease following COVID-19 infection have been previously reported [5, 6], with 57.1% of patients who developed new-onset uveitis following COVID-19 vaccination being diagnosed with VKH [12]. However, this is not unexpected, as viruses and vaccines are thought to trigger an immune response, especially in genetically susceptible individuals, driving VKH disease pathogenesis [13–15].

The impact of COVID-19 infection on patients with a history of uveitis is a pressing concern among uveitis experts. However, to date, no studies have directly investigated the effect of COVID-19 infection on uveitis relapse rates. Moreover, some studies have investigated the link between uveitis relapse and COVID-19 vaccination, but the results are contradictory. For example, Song et al. [16] found no statistical difference in the uveitis relapse rate between vaccinated and unvaccinated individuals 30 or 60 days post-vaccination. In contrast, a previous study reported uveitis relapse rates of 12.3 and 20.7 per 1,000 patient-months, three months before and after vaccination, respectively, suggesting an increased risk of recurrence after the first dose of the vaccine [17]. Similarly, data on the impact of COVID-19 infection on multiple sclerosis (MS), a systemic autoimmune disease, are also inconsistent, with Barzegar et al. [18] reporting that COVID-19 infection can worsen MS and Etemadifar et al. [19] reporting no effect.

To ensure a comparable study context, we compared levels of inflammation between COVID-19-infected and non-infected patients during the same follow-up period. COVID-19 infection increased the risk of uveitis relapse; however, determining whether there was a direct causal relationship is challenging. The relapse risk varies across studies owing to several factors, including differences in follow-up times. In relapsing-remitting diseases, studying relapse rates within 5 weeks and 6 months post-infection may result in variability, with longer follow-up periods tending to diminish the observable impact of COVID-19 infection. Second, differences in patient clinical characteristics may contribute to variable outcomes; for example, variability in levels of inflammation among patients in different studies can lead to divergent results. Finally, the sample size is an important consideration, as underpowered studies may fail to detect an effect.

In the present study, we initially investigated the risk factors associated with uveitis relapse post-COVID-19 infection; a longer disease duration, non-use of methotrexate during COVID-19 infection, and a previous relapse episode occurring within one year prior to COVID-19 infection were independent risk factors. The duration of uveitis is an indicator of disease severity, with longer durations increasing the likelihood of recurrence and irreversible structural damage to the eye [20–22]. In VKH disease, longer periods since disease onset contribute to poorer control of uveitis, leading to recurrent episodes of granulomatous uveitis [23].

In terms of the effects of methotrexate, Schälter et al. [24] noted that its protective effect against the SARS-CoV-2 virus occurred via the downregulation of angiotensin-converting enzyme 2. In a study of COVID-19-related mortality factors in patients with rheumatic diseases, the use of rituximab, sulfasalazine, and other immunosuppressive agents was associated with higher COVID-19-related mortality than methotrexate monotherapy [25]. In a comparative study, methotrexate did not predispose patients to severe COVID-19; instead, it tended to result in milder disease severity [26]. The antiviral effect of methotrexate may be due to the inhibition of TNFα, decrease in IL6 levels, and increase in T regulatory cell levels, leading to a reduction in severe inflammatory responses, and is associated with its inhibitory effect on specific protein-protein interactions [26–28]. Our current study showed that patients who were administered methotrexate during their COVID-19 infection had a reduced risk of relapse of uveitis. It has been hypothesized that SARS-CoV-2 can disrupt self-tolerance and stimulate autoimmune responses through molecular mimicry and its cross-reactivity with host cells [29, 30], and viruses can activate ocular Toll-like receptor 4 (TLR 4) to initiate innate immune responses [31, 32]. Our study provides evidence that methotrexate inhibits uveitis relapse by suppressing immune responses associated with these processes. Collectively, these data suggest that methotrexate is a safe option for patients with uveitis, as it can attenuate the cytokine storm associated with COVID-19 infection and prevent uveitis relapse. However, the present study used fewer other types of immunosuppressive agents. Therefore, further studies are required to determine the effect of using different immunosuppressive agents on uveitis relapse following COVID-19 infection.

Longer periods of uveitis inactivity have been shown to be associated with lower reactivation rates [17], which is consistent with our finding that duration since the last relapse episode of more than one year prior to COVID-19 infection was a significant protective factor against uveitis relapse. This indicated that regular follow-up visits, standardized medication use, and sustained inflammatory control measures significantly decreased the likelihood of uveitis relapse. Notably, delaying the administration of medications for uveitis during COVID-19 infection did not affect uveitis relapse rates. Due to an emphasis on avoiding arbitrary discontinuation of corticosteroids during weekday treatment to prevent rebound phenomena, the postponed systemic drugs for uveitis treatment primarily included adalimumab and immunosuppressive agents. Immunosuppressive agents exert anti-inflammatory effects by suppressing immune cell production and most immunomodulatory therapies require several weeks to take effect [33]. Consequently, even a brief delay in the use of immunosuppressive agents does not immediately restore the production of suppressed immune cells. Adalimumab, an anti-TNF-α monoclonal antibody, is typically injected subcutaneously every 2 weeks, and the onset phase of COVID-19 infection typically occurs within 14 days. Therefore, a short delay in administering the injection does not significantly affect relapse risk. However, the results must be interpreted cautiously for several reasons. First, in the present study, > 80% of patients had grade 0 for anterior chamber cells during their last visit prior to COVID-19 infection, indicating relatively stable inflammation. Second, for patients experiencing different active stages of uveitis, the decision to halt systemic medications during COVID-19 infection should be determined comprehensively by the physician, given that most patients have already been vaccinated against COVID-19.

This study had several limitations. First, it is an observational study conducted at a single center, which restricts exploration regarding the incidence or risk of new-onset uveitis after COVID-19 infection in a broader population. Additionally, the number of patients with new-onset anterior uveitis was limited because patients with milder disease are less frequently referred to level 3 treatment centers. Second, this study only evaluated uveitis development post-COVID-19 infection within a specific period. The time interval of 2 months post-infection was selected to capture patients with a long latency period and those who were not seen in time as a result of not exhibiting obvious spontaneous symptoms. However, a longer time interval may weaken the temporal relationship between COVID-19 infection and uveitis. Additionally, most of the Chinese population had already been vaccinated against COVID-19 at the time of the study, which could have impacted subsequent immune system changes and the body’s response to COVID-19 infection. Finally, we also lacked data on other systemic medications administered during COVID-19 infection, including drugs used to treat COVID-19 itself. Therefore, further research is required to examine the effects of these medications on uveitis.

## Conclusions

Our study demonstrates that COVID-19 infection is associated with an increased uveitis relapse rate. Additionally, we identified several risk factors for post-COVID-19 infection, which include a longer disease duration, the non-use of methotrexate during COVID-19 infection, and a last relapse episode occurring within one year before COVID-19 infection. Thus, the likelihood of uveitis relapse may be higher in such patients, and they should promptly seek medical attention to allow for close monitoring of inflammation post-COVID-19 infection.

## Data Availability

The data and materials used in this study are available from the corresponding author on reasonable request.
